# Trajectories of social withdrawal and social anxiety and their relationship with self-esteem before, during, and after the school lockdowns

**DOI:** 10.1038/s41598-023-43497-w

**Published:** 2023-09-29

**Authors:** Sara Cruz, Mariana Sousa, Marta Marchante, Vítor Alexandre Coelho

**Affiliations:** 1 Department of Psychology, School of Philosophy, Psychology & Language Sciences, University of Edinburgh, Edinburgh, UK; 2 Psychology for Development Research Centre, Lusíada University, Porto, Portugal; 3 Académico de Torres Vedras, Torres Vedras, Portugal

**Keywords:** Human behaviour, Psychology

## Abstract

The closure of schools during the COVID-19 pandemic affected adolescents’ social withdrawal and social anxiety. Yet, self-esteem may have acted as a protective factor during this period. This study aimed to compare the trajectories of social withdrawal and social anxiety before (Year 1), during (Year 2 and 3), and after (Year 4) the closure of schools imposed by the COVID-19-related lockdowns, and to investigate the association of self-esteem with these trajectories. Participants were 844 (50.6% boys) Portuguese adolescents (mean age 12.70 years, *SD* = 1.14). The Social and Emotional Competencies Evaluation Questionnaire (QACSE) was used to assess social withdrawal and social anxiety, while The Global Self-Esteem scale of the Self-Description Questionnaire II was used to measure self-esteem. Growth curve analysis showed that social withdrawal and social anxiety had more negative trajectories during the year in which the school closures occurred. In addition, adolescents reported higher social withdrawal after the lockdowns than before the pandemic. Higher self-esteem was associated with a more positive trajectory in social withdrawal. Therefore, the results showed the negative of impact of the closure of schools on adolescents’ social anxiety and social withdrawal, and that self-esteem was a protective factor during these challenging and adverse events.

## Introduction

The global pandemic of coronavirus disease 2019—COVID-19—has negatively affected the mental health of the general population, as environmental stressors constitute one of the main etiological factors of most mental disorders^1^.

At the onset of the pandemic, governments around the world implemented measures to control and mitigate the dissemination of the COVID-19 disease, namely lockdowns, quarantine, isolation, and social distancing. In Portugal, the first lockdown was decreed on 18 March 2020. During this period, schools closed and, one month later, distance learning was adopted. On 4 May 2020, Portugal started easing restrictions and, consequently, high schools reopened. But middle schools remained closed. The second lockdown was implemented on 15 January 2021 (due to the large number of individuals affected by the COVID-19 disease), and schools were closed again. This closure lasted until March. However middle school students were only allowed to return to school on 19 April 2021.

These measures radically altered daily routines and social interactions. This disruption and unpredictability resulted in severe distress and acted as a risk factor for increased levels of social anxiety^2^. Adolescents were particularly vulnerable to these changes, as school is possibly their most important social setting, having a significant impact on their development and adjustment^3^. The closure of schools significantly altered adolescents’ social interactions, with reports of increased social isolation or loneliness, often leading to the emergence of symptoms of social anxiety^4^. Interestingly, recent evidence indicates that even after returning to their normal routines, with COVID-19-related prevention measures and policies being revisited and lifted worldwide, adolescents are among those who still report debilitating effects of the pandemic, indicating continued high levels of anxiety^5^. It is therefore important to clarify the impact of these measures on adolescents’ social withdrawal and social anxiety, and their relationship with self-esteem, by comparing how students’ trajectories of social anxiety and social withdrawal varied before, during, and after the closure of schools as a result of the pandemic-related lockdowns.

### Social withdrawal and social anxiety and the effect of the lockdowns

Social withdrawal is a multidimensional, behavioral phenotype of voluntary self-isolation^6^. This phenomenon is expressed by the avoidance of social interactions, regardless of the individual’s familiarity with them, and by remaining alone at higher rates in social gatherings compared to their age mates^7,8^. The motivations behind social withdrawal may be due to (i) the need to be alone; (ii) the need to isolate oneself from peers in social interactions and/or actively choose to be alone to avoid initiating and maintaining interpersonal relationships; and (iii) isolation or rejection by others in their significant contexts^8^.

Social withdrawal is part of adaptive functioning, as withdrawn adolescents intentionally isolate themselves and do not experience any discomfort or distress from spending too much time alone or avoiding social interactions^9^. However, it may also reflect the presence of social and/or emotional vulnerabilities, which act as psychopathological markers, such as social isolation and/or rejection^10^. These adolescents often lack important social opportunities to promote their positive development and may not be accepted by their peers due to their atypical behaviors^11^. Avoiding exposure to social situations also limits the development of social skills, which would be helpful in increasing their sense of self-efficacy when dealing with interpersonal challenges^11^.

Although they are different constructs, social withdrawal is closely linked to social anxiety. Social anxiety refers to the severe and pervasive fear of engaging in social interactions, caused by the worry of being judged and criticized by others, unduly evaluated, or even rejected^12^. Social anxiety can be framed on a continuum between adjusted responses of social concern and severe and persistent fear associated with avoidance of social interactions^13,14^. This more intense social fear observed at the end of the continuum impairs social functioning, resulting in a cycle of social avoidance^13,14^. Thus, individuals with severe social anxiety may not be able to establish social connections, due to their bias in reading social situations, which tend to be perceived as threatening^15^.

The onset of social anxiety is usually in adolescence and is one of the most prevalent forms of anxiety in this developmental period^12,16^. Because of their increased autonomy, adolescents are able to expand their social network, which is no longer exclusively dependent on parents, and peers become a central element of this network, acting as a source of identity and approval^17^. As peer relationships are an essential source of social support for adolescents, difficulties in coping with peer interactions are often related to maladaptive outcomes, namely anxiety symptoms, but also depressive symptoms, along with externalizing behaviors^18^.

However, it is important to note that despite the close association between social withdrawal and social anxiety, as they influence each other both concurrently and longitudinally, these dimensions do not categorically overlap^6,8^. Evidence shows that although higher levels of social withdrawal or social anxiety may increase the vulnerability to the other, not all withdrawn adolescents become more socially anxious, and not all socially anxious adolescents become more withdrawn^6,7^.

Forced social avoidance (e.g., resulting from the COVID-19 related lockdowns) tended to intensify social anxiety both in clinical and nonclinical populations^19,20^**,** and social activities were identified as a primary concern among adolescents (e.g., reduced opportunities to see friends)^21^. Yet, different trajectories for anxiety were observed, as adolescents who exhibited higher levels of pre-pandemic stress, maladaptive coping mechanisms, internalizing problems, and limited social support were at increased risk of experiencing higher anxiety levels during the pandemic compared to those without these pre-existing vulnerabilities^21,22^. Moreover, an overarching trend of increasing concerns persisted throughout the pandemic, with those related to social activities and contamination remaining relatively stable in the post-pandemic period^21^.

The limited face-to-face interactions caused by the pandemic provided adolescents who previously dealt with social anxiety a greater sense of control, as they perceived their exposure to social situations as more effective, namely by restricting their social interactions to only those with whom they feel most safe and secure^23,24^. Nevertheless, they continued to use maladaptive coping strategies to manage their anxiety, such as self-monitoring and safety behaviors (e.g., controlling the personal information provided, managing the time spent in writing a response, and turning-off the camera to avoid their visual exposition)^25^. Adolescents were also prevented from establishing adequate social connections, and their access to social support was significantly diminished^26^. This social deprivation reinforced social avoidance behaviors, increasing their anxiety levels^2,20^, and making the return to school quite difficult for those who felt protected from public exposure during lockdowns^23^.

### Self-esteem, social withdrawal, and social anxiety

Self-esteem refers to a person’s positive or negative judgment and attitudes about their competence and sense of worth^27,28^. It is a global component of self-concept^29^, constituting its descriptive and evaluative dimension, as demonstrated by statements such *I am great at fine arts* or *I have charisma* show^30^. Self-esteem is anchored in cognitive and affective components^31^, referring to the perceptions that individuals create based on their experiences and interpretations of the environmental cues^32^, such as the others’ evaluations and reinforcements, and attributions for their own behavior, and predicts future behavior^31^.

It is well established in the literature that lower levels of self-esteem increase vulnerability to the development of social anxiety symptoms, as goal setting to validate individual abilities and self-worth are at the center of both social anxiety and self-esteem^28,33^. This is largely because adolescents’ self-esteem development depends mostly on the relationships established with their peers and how they are evaluated by them^34^. Individuals who perceive and evaluate themselves negatively tend to avoid social interactions or be passive in social encounters, lack confidence in interpersonal situations, and fear social scrutiny, which in turn increases their social anxiety^12^. Similarly, individuals with low self-esteem often expect future experiences to be negative and use negative coping strategies to deal with unpredictable situations (e.g., avoidance, procrastination, or self-handicapping), which also increases social anxiety^33^.

Low levels of self-esteem are also associated with greater social withdrawal, as withdrawn individuals frequently isolate themselves from peer groups due to difficulties in dealing with social interactions^8^. Social withdrawal hinders the development of social skills, and poor social skills tend to reinforce social anxiety and promote negative self-appraisals, thus negatively impacting self-esteem^35^. A recent systematic review showed that social connectedness (i.e., connection to school) and self-esteem were among the most common protective factors for social isolation after periods of lockdown due to pandemic-related control measures^36^.

Additional evidence suggests that self-esteem may act as a moderator factor for social withdrawal and social anxiety^37^. Yuan et al.^37^ observed an effect of low self-esteem levels in the association between interpersonal trust and social avoidance, as the interaction between interpersonal trust and self-esteem had a significant negative predictive effect on social avoidance. According to the authors, individuals with low self-esteem are more likely to be affected by the effect of interpersonal trust on social avoidance, as they are poorly skilled in monitoring the degree of acceptance and exclusion by others and using strategies to reduce the possibility of being rejected. Moreover, other findings indicate that even moderate levels of self-esteem seem to decrease the association between loneliness and peer relationship quality in adolescents^38^. Therefore, self-esteem possibly has a buffering role which decreases the negative effect of loneliness on social anxiety, acting as a moderator factor in this association. During the pandemic, self-esteem was one of the most important protective factors against social isolation^36^. Similarly, lower self-esteem appears to contribute to anxiety symptoms, while higher self-esteem may mitigate them^39^.

### The current study

Considering the aforementioned evidence, it is important to clarify how the trajectory of students’ social withdrawal and social anxiety was affected during the COVID-19 pandemic. Specifically, this study aimed to compare the trajectory of social withdrawal and social anxiety, before, during and after the closure of schools, due to the COVID-19-related lockdowns, in a sample of Portuguese adolescents. This study also analyzed how self-esteem was associated with the trajectories of social withdrawal and social anxiety in adolescents in this period.

We hypothesized that students’ social withdrawal (H1) and social anxiety (H2) had a more negative trajectory in school years when lockdowns occurred (i.e., schools were closed—Year 2 2019/2020 and Year 3 2020/2021) compared to school years when schools were open (i.e., before—Year 1 2018/2019—and after—Year 4 2021/2022—the lockdowns), leading to an increase both in social withdrawal and social anxiety. Moreover, given the theoretical background presented before, it is likely that self-esteem has a protective effect, particularly during critical events, such as the closure of schools. Thus, we also hypothesized that (H3) in the school years in which schools were closed (i.e., Year 2 2019/2020 and Year 3 2020/2021), students’ higher self-esteem will be associated with more positive trajectories in their social anxiety and social withdrawal compared to the school year prior to lockdowns and the closure of schools (Year 1 2018/2019), contributing to reduce these two dimensions**.**

## Method

### Participants

The sample for this study was collected as part of the Gulbenkian Academies of Knowledge (GAK), a nationwide dissemination initiative of Social and Emotional Learning (SEL) blueprint programs in Portugal. The sample only includes the control groups of the SEL intervention. Participants were 844 upper middle school students (7th and 8th grade, at the time of the first assessment), from 40 classes of 14 public schools of Continental Portugal and the Madeira Archipelago. The sample is gender-balanced, with 427 boys (50.6%) and 417 girls (49.4%). The participants’ mean age was 12.70 (*SD* = 1.14). Schools varied considerably regarding socioeconomic status (i.e., the percentage of students eligible for free or reduced school meal ranged from 22.3 to 74.0%) and classrooms varied regarding ethnicity (i.e., between 1 and 18% of students were descended from minorities in Portugal). Therefore, socioeconomic status and ethnicity were included as variables in the models. Table 1 provides additional information about the participants.

**Table 1:** Self-reports—descriptive statistics for self-esteem, social anxiety and social withdrawal across times, per gender and year.

	N = 844	Participants						
		Time 1			Time 2		Time 3	
		*n* = 837			n = 817		n = 794	
		Self-esteem *M* (*SD*)	Social anxiety *M* (*SD*)	Social withdrawal *M* (*SD*)	Social anxiety *M* (*SD*)	Social withdrawal *M* (*SD*)	Social anxiety *M* (*SD*)	Social withdrawal *M* (*SD*)
Gender								
Boys	n = 427	38.82 (7.07)	6.77 (3.53)	4.72 (3.42)	7.34 (3.76)	5.25 (3.65)	7.52 (3.52)	5.41 (3.43)
Girls	n = 417	36.35 (8.52)	9.98 (4.22)	5.39 (3.86)	10.09 (4.06)	5.74 (4.10)	10.26 (3.84)	5.96 (3.71)
		t(835) = 4.56***	t(835) = − 11.94***	t(835) = − 2.64**				
Year								
Year 1 2018/2019	n = 175	38.20 (7.08)	8.18 (3.87)	4.31 (3.47)	8.33 (4.15)	4.16 (3.40)	8.04 (3.64)	4.14 (2.95)
Year 2 2019/2020	n = 184	37.37 (8.11)	8.80 (4.25)	4.92 (3.69)	9.21 (3.76)	5.65 (3.68)	9.36 (3.71)	5.58 (3.45)
Year 3 2020/2021	n = 270	37.85 (7.61)	7.92 (4.20)	4.84 (3.30)	8.47 (4.29)	5.55 (3.66)	9.00 (4.11)	6.16 (3.49)
Year 4 2021/2022	n = 215	35.68 (8.45)	8.70 (4.27)	6.06 (4.01)	8.86 (4.26)	6.39 (4.40)	8.99 (4.00)	6.46 (3.89)
		F(3,833) = 6.88***	F(3,833) = 2.21	F(3,833) = 8.33***				

*p < 0.05; **p < 0.01; ***p < 0.001.

Regarding attrition, eleven parents (1.3%) did not consent to their youths’ participation in the assessments, and these students were not included in the sample described above. Eight-hundred-thirty-seven seventh and eighth-grade students (99.2%) participated in the first assessment, 817 (96.8%) in the second assessment, and 794 (94.1%) in the third assessment. Twenty students changed schools between the two first assessments, decreasing the number of participants enrolled in the second moment. Another 24 students did not participate in the third assessment because they either changed schools or were retained in the same grade at the end of the previous school year and were no longer part of the same class.

### Measures

#### Social withdrawal and social anxiety

The Social and Emotional Competencies Evaluation Questionnaire (QACSE^40,41^) was used to assess social withdrawal and social anxiety. Both social withdrawal (e.g., “I isolate myself and do not talk to anyone”; α = 0.74; α = 0.77 in the present study) and social anxiety (e.g., “I get afraid when I face new situations”; *α* = 0.78; α = 0.84 in the present study) subscales comprise seven items that are rated on a four-point Likert scale (0 = *never* to 3 = *always*). Two different studies were conducted to validate the QACSE with adolescents (11 to 16 years). In the first one^40^, involving 683 adolescents, the instruments’ factorial structure was established, whereas in the second study^41^, with 655 adolescents, a confirmatory factorial analysis was conducted, supporting the factorial structure used.

#### Self-esteem

The Global Self-Esteem scale (Portuguese version^42^ of the Self-Description Questionnaire II^43^ was used to assess self-esteem. This scale measures global self-worth based on ten items (e.g.: “I am successful in doing most of the things I do.”) assessed in five-point Likert scale (1 = *false* to 5 = *true*). To determine the total score, the scores for five questions must first be reversed, as they are presented as negative statements. Then, the scores for each item are summed up to obtain the total score. The internal consistency of the scale in both the original version (α = 0.88) and the Portuguese adaptation (α = 0.82) is good.

#### School and classroom characteristics

Official school records were used to gather information on school and classroom characteristics (i.e., free and reduced school meals, and classroom size).

### Procedure

This study is part of a larger project currently conducted at The Psychology for Development Research Center of Lusíada University (CIPD/2122/DSE/2) and it was reviewed and approved by the Ethics Committee of Lusíada University (approval reference UL/CE/CIPD/2308). Informed consent was obtained from all the study participants through forms sent to parents and adolescents at the beginning of the school year. The national ethical principles and code of conduct, which provides guidance for Portuguese psychologists’ professional practice, along with the national legislation, were also considered when designing this study.

The questionnaires were administered to middle school students in three different moments: T1 (at the beginning of each school year—October)—baseline assessment of social withdrawal, social anxiety, and self-esteem; T2 (at the end of the first semester of the school year—February)—mid-year assessment of social and social withdrawal and social anxiety; and T3 (at the beginning of the next school year)—follow-up assessment of social withdrawal and social anxiety. Data were collected throughout four different school years: Year 1 (2018/2019)—before the pandemic; Year 2 (2019/2020) —first lockdown and closure of schools; Year 3 (2020/2021)—second school lockdown and closure of schools; and Year 4 (2021/2022)—after the lockdowns and schools reopened.

The questionnaires were filled out electronically using school computers or tablets—supplied by the GAK initiative—in an online platform designed for this purpose, thus resulting in no missing data at the individual level. The assessments were performed by the same educational psychologist in all assessment moments. The psychologist explained the instructions during regular scheduled classes in the presence of a teacher. Completing the questionnaires lasted around 20 min. If a student was not present during the data collection, the psychologist returned the following week (n = 49).

### Data analyses

First, to provide support for the reliability of the measures used, the Cronbach’s α index was calculated using with the study’s sample. Then, we conducted *t*-tests to compare students’ initial self-esteem, social anxiety, and social withdrawal between genders, and one-way ANOVAs to compare the initial levels of self-esteem social anxiety and social withdrawal between school years.

To test the current study’s hypotheses, we performed multilevel linear modeling using MLwiN 2.36. We selected this analytical strategy given the hierarchical and clustered nature of the study data set, as students from the same classroom are likely to provide highly correlated responses^44^. This option is also in line with Bliese et al.^45^, who suggested that low ICC values for level-3 predictors should not discourage researchers from using multilevel linear modeling. Thus, three-level models were conducted, as the three assessments were nested within the 844 students, which were nested within 40 school classrooms. Model fitness was assessed through the comparison of the function of log-likelihood, using a model deviance test to compare the log-likelihoods. Specifically, model fit is better when the difference between models is statistically significant after adjusting for the differences in degrees of freedom, i.e., the second model is significantly smaller than the previous one.

To conduct these analyzes, we first assessed the normality assumptions, by checking the distribution of residuals at all three levels with normal probability plots. Straight-line plots of generated normal scores against the standardized residuals showed normally distributed residuals. A series of models were created for both outcomes (these are available in the Supplemental materials). All models employed full information maximum likelihood and all models used the intercept as a random effect. First, an unconditional model (Model 0) that included no predictors was used to analyze between-classrooms variance, creating Model 1. This model was a growth curve model in which the effect of continuous time on the outcome was treated as linear and quadratic (if needed) and allowed to vary across individuals or classrooms (random slope) to assess within-individual variation. Next, gender and initial self-esteem were entered as explanatory variables at the individual level (Model 2). For Model 3, free and reduced school meals and ethnicity (both grand-mean centered), and school year of assessment (Year 1 vs. Year 2 vs. Year 3 vs. Year 4) were entered as explanatory variables at the class level. Model 4 comprises the cross-level interaction between Year^*^Time to test hypothesis one and two. In the final models (i.e., model 3 and 4), two three-way cross-level interactions terms (one for social anxiety and another for social withdrawal) were specified to test hypothesis three. These cross-level interactions included Time^*^Self-Esteem^*^Year.

## Results

### Self-esteem, social withdrawal and social anxiety before, during, and after the closure of schools

Table 1 shows the descriptive statistics for self-esteem, social withdrawal, and social anxiety across timepoints, organized by gender and year. At Time 1, girls reported higher social withdrawal and social anxiety, and lower self-esteem than boys. When comparing scores by year, statistically significant differences emerged in self-esteem and social withdrawal (cf., Table 1). Post hoc comparisons, using the Tukey HSD test, indicated that students from the Year 4 (i.e., after the pandemic lockdowns and thus the closure of schools) presented significantly lower mean scores (*p* = 0.010) for self-esteem compared to those from the Year 1 (i.e., before the pandemic). Post hoc comparisons also revealed that Year 4 students showed higher levels of social withdrawal compared to those from all the other years—Year 1 (*p* < 0.001); Year 2 *(p* = 0.11); and Year 3 (*p* = 0.001).

### Trajectories of social withdrawal and social anxiety

This study aimed to analyze differences in the trajectories of social anxiety and social withdrawal before, during, and after the closure of schools due to the lockdowns imposed by the COVID-19-related measures to control its dissemination. Table 2 presents the results of the final models for social anxiety and social withdrawal (all previous models for social anxiety are available in Supplemental Table 1 and for social withdrawal in Supplemental Table 2).

**Table 2:** Multilevel model analysis final models for social anxiety and social withdrawal.

	Social anxiety		Social withdrawal	
	*β*_0*ijk*_ = 9.67 (0.29)***		*β*_0*ijk*_ = 4.4 (0.26)***	
	Co-efficient β	SE	Co-efficient β	SE
Classroom				
Free and reduced school meals	1.36	1.29	2.24	1.25
Ethnicity	0.51	1.94	− 0.77	1.88
Year (if year 2)	0.41	0.37	0.46	0.33
Year (if year 3)	− 0.14	0.35	0.60	0.31
Year (if year 4)	− 0.02	0.36	1.23***	0.32
Student				
Gender (if boys)	− 2.63***	0.24	0.06	0.20
Self-esteem	− 0.20***	0.02	− 0.26***	0.01
Time				
Time linear	− 0.15	0.12	− 0.07	0.11
Interactions				
Gender (if boys) × time linear	− 0.21*	0.10	− 0.03	0.09
Year (if year 2) × time linear	0.35*	0.15	0.45**	0.14
Year (if year 3) × time linear	0.60***	0.14	0.75***	0.13
Year (if year 4) × time linear	0.23	0.15	0.24	0.14
Time × self-esteem × year (if year 2)	0.01	0.01	0.04***	0.01
Time × self-esteem × year (if year 3)	0.01	0.01	0.01	0.01
Time × self-esteem × year (if year 4)	0.01	0.01	0.01	0.01
Repeated measures	3.270***	0.164	3.197***	0.114
Individual intercept	8.991***	0.590	5.963***	0.357
Individual slope	0.392**	0.131		
Individual covariance intercept/slope	− 0.364	0.203		
Classroom intercept	0.011	0.016	0.034	0.099
Classroom slope			0.008	0.019
Classroom covariance intercept/slope			0.095**	0.031
*Deviance* (−2_loglikelihood_)	11,787.890		11,317.364	
Estimated parameters	21		21	

*p < 0.05; **p < 0.01; ***p < 0.001.

Regarding social withdrawal, after adjusting for all individual and classroom-level variables, as well as cross-level interactions, time was no longer a statistically significant predictor. Social withdrawal did not vary significantly independently of other predictors in the model in the analyzed period. Among the individual level variables, only self-esteem was a statistically significant predictor of social withdrawal, in that students with higher self-esteem reported lower social withdrawal. Regarding the classroom level variables, only being a Year 4 student was a statistically significant predictor of social withdrawal. Year 4 students reported significantly higher social withdrawal compared to Year 1 students. Furthermore, two statistically significant two-way cross-level interactions were observed—between Year 2 and time, and between Year 3 and time. Year 2 and Year 3 students reported more pronounced increases in social withdrawal in the analyzed period, thus supporting hypothesis one.

As for social anxiety, after adding all predictors and cross-level interactions, the within-individual variable ‘time’ was not a statistically significant predictor. Social anxiety did not vary significantly independently of other predictors in the model in the analyzed period. The other statistically significant predictors of self-reported social anxiety were gender, self-esteem, and three two-way cross-level interactions, between gender and time, between Year 2 and time, and between Year 3 and time. Boys reported both lower social anxiety and a more pronounced decrease in their social anxiety than girls. Whereas students with higher self-esteem reported lower social anxiety. In addition, Year 2 and Year 3 students reported more pronounced increases in social anxiety in the analyzed period, thus supporting hypothesis two.

### The association between self-esteem and social withdrawal and social anxiety

Equally, this study aimed to analyze if students’ higher self-esteem (assessed in Time 1) was associated with more positive trajectories in their social anxiety and social withdrawal when schools were closed (Year 2 and Year 3) compared to when schools were open (Year 1). To test these hypotheses, a series of three-way cross-level interactions between time, self-esteem (grand-mean centered) and Year (Year 1 vs. Year 2 vs. Year 3 vs. Year 4) were added to the final models. For social anxiety, none of the three-way cross-level interactions between time, self-esteem, and year produced statistically significant results (cf., Table 2), thus partially negating hypothesis three. On the contrary, higher self-esteem were associated with smaller increases in social withdrawal during the analyzed period, *β* = 0.04, SE = 0.01; *z* = 3.82, *p* < 0.001, partially supporting hypothesis three.

## Discussion

The pandemic environment has imposed structural social changes, mainly due to the measures implemented by governments worldwide to prevent the spread of the COVID-19 disease (i.e., government-imposed lockdowns, social distancing, or restrictions of social interactions). In particular, the closure of schools raised significant concerns as it has severely affected adolescents’ mental and social functioning^46^. This study investigated the trajectories of social withdrawal and social anxiety before, during, and after the imposition of COVID-19-related lockdowns, and the consequent closure of schools, in a sample of Portuguese adolescents. Moreover, it examined the relationship between self-esteem and the trajectories of social withdrawal and social anxiety.

As expected, the trajectories of social withdrawal and social anxiety were worse during the pandemic, with the results showing that, in this period, adolescents reported higher levels of social withdrawal and social anxiety. This is in line with other evidence that has demonstrated the negative impact of the imposed lockdowns on adolescents’ mental health^47^. The disruption of social relationships caused by the closure of schools and social distancing has hampered adolescents’ social functioning and their ability to establish meaningful social bonds, perhaps leading to a perceived lack of support and chronic loneliness^6,11,48^. These radical changes in social routines may have also reinforced the avoidance of exposure to social situations, along with the use of maladaptive coping strategies to manage anxiety, such as self-monitoring and safety behaviors, thereby increasing both social withdrawal and social anxiety^25^. This may have reduced adolescents' perceived efficacy in dealing with interpersonal interactions^11^ and increased social isolation and/or rejection associated with social and emotional problems^10^^.^

When examining the specific trajectories of social withdrawal and social anxiety, distinct factors significantly contributed to changes in these dimensions when comparing the periods before, during and after the closure of schools due to the lockdowns.

Considering social withdrawal, adolescents reported significantly higher experiences of social withdrawal after the lockdowns (i.e., schools were reopened as a result of the government lifting lockdowns), compared to the period before the pandemic. This increase may be related to the forced periods of social distancing, and consequent feelings of loneliness, imposed by lockdowns and the closure of schools^5^. Indeed, evidence shows that after critical events, such as the pandemic, young people continue to report higher depressive and anxiety symptoms than adults, which may be due to the disruption of adolescents’ normative social development driven by extreme circumstances, such as the closure of schools, which has been linked to social withdrawal^2^. Moreover, with school possibly being their most important social context, adolescents were particularly vulnerable to these changes^3^.

Our results show that gender seems an important factor for the trajectory of social anxiety, as boys reported lower social anxiety and a more pronounced decrease in their social anxiety during COVID-19 pandemic, compared to girls. One possible explanation may be that even before the pandemic, girls appear to be particularly vulnerable to experience social anxiety^49^, and to report lower self-esteem than boys^50^. This suggests that girls may already start at a disadvantage in coping with pandemic-related stress. This is consistent with other research showing that girls experience higher symptoms of depression and anxiety than boys during the lockdowns, with concerns predominantly related to school^35^. It is possible that girls tend to exhibit an intensified stress response, as a large body of research shows that they are more likely to develop internalizing problems than boys, which is even more prominent in atypical circumstances^51,52^.

As expected, adolescents who reported higher self-esteem also stated lower social anxiety. As self-esteem is a natural buffer to reduce anxiety levels^53^, it is possible that it helps adolescents to deal more effectively with atypical situations (e.g., closure of schools and social distancing) that impose disruptions to social connections and interactions^54^. However, we observed that adolescents reported lower levels of self-esteem after the pandemic lockdowns compared to the period before the pandemic. It is possible that the lack of social connection, due to the closure of schools, may have affected adolescents’ sense of worth, as adolescence is a particular important period to develop their identity among peers and in society^17^. Thus, limited school and social activities appeared to be detrimental to the youth’s self-esteem, as social isolation is associated with greater mental health problems at this age period^55^.

Nevertheless, we observed that self-esteem was a protective factor during the pandemic but only for social withdrawal. Students who indicated higher self-esteem also reported a more positive trajectory in social withdrawal. Self-esteem is an intra-individual factor that seems to protect adolescents from mental health issues during unpredictable situations^56^. In alignment with other evidence, our results indicate that self-esteem seemed to have acted as positive coping mechanism for young people to deal with stressful situations, particularly with social distancing, and mediated the negative impact of the pandemic lockdowns on mental health^57^. This effect was not observed for social anxiety, as lockdowns may have acted as a negative reinforcement, discouraging social situations, and significantly reducing adolescents’ anxiety levels. Self-esteem possibly played a secondary role in this situation. For example, one study showed that adolescents with high pre-pandemic social anxiety experienced lower levels of anxiety and worry during the pandemic^58^. It is possible that those who reported higher pre-pandemic levels of social anxiety benefited greatly from the closure of schools, as they were not exposed to anxiety-provoking situations in the school environment^58^.

In sum, the social distancing policies imposed by the COVID-19 pandemic have had a negative impact on individuals’ mental health (e.g., increased social withdrawal and social anxiety). This situation seems to have been particularly aggravated for adolescents who experienced the closure of schools and, consequently, had limited socialization with their peers, and spend much less time outdoors. It is therefore important to continue investigating multi-level risk and protective factors, as contexts affected by the pandemic can have a chronic impact on mental health, especially for adolescents, who need tailored intervention programs.

This work offers multiple strengths, including the analysis of the contributions of the lockdowns and closure of schools to social withdrawal and social anxiety in adolescents, as these results can provide information for intervention approaches in this population. Our results highlight the need to support adolescents at risk of mental distress in times of increased stress and uncertainty, possibly contributing to fostering greater resilience in challenging situations. It is crucial that interventions with these adolescents focus not only on visible symptoms or maladaptive behaviors associated with social anxiety, but also prioritize dimensions that are fundamental to their psychological functioning, such as self-esteem. Merely helping adolescents develop strategies to cope more effectively with social encounters and reduce anxiety levels when facing social exposure may be insufficient to enhance their resilience when facing adversity.

However, this study has some limitations. A longer longitudinal study would be able to better determine the causal associations between self-esteem, social anxiety, and social withdrawal. Thus, futures studies should continue to determine whether the effects of the pandemic are temporary or stable changes that characterize this generation of adolescents, using longitudinal approaches. Additionally, evidence points to a bidirectional and cyclical relationship between social withdrawal and social anxiety^8,9^, which was not considered in this study. Therefore, it is important to address this relationship in the context of the pandemic, as well as its impact on mental health and other developmental outcomes (e.g., social and emotional competencies).

### Supplementary Information

Supplementary Tables.

## Data Availability

The data that supported the findings of this study is available upon request to the corresponding author. The data is not publicly available due to privacy or ethical restrictions.
