# Supplementary material for: Trajectories of social withdrawal and social anxiety and their relationship with self-esteem before, during, and after the school lockdowns
Source: Sci Rep. 2023 Sep 29;13:16376. doi: 10.1038/s41598-023-43497-w (PMC10542336; doi:10.1038/s41598-023-43497-w)
Supplement: Supplementary file 1 — Supplementary Tables. [file 41598_2023_43497_MOESM1_ESM.docx]

Supplementary Table 1

*Multilevel Model Analysis Models for Social Anxiety*

| Parameters | Model 0  Null | Model 1  Level 1: Time |  | Model 2  Level 2: Individual | Model 3  Level 3: Class | Model 4  Cross-Level Interactions  Level 1 and 3 |
| --- | --- | --- | --- | --- | --- | --- |
| Estimates of Fixed Effects | | | | | | |
| Intercept | 8.64 (0.15)^***^ | 8.38 (0.16)^***^ |  | 9.60 (0.17)^***^ | 9.24 (0.27)^***^ | 9.67 (0.29)^***^ |
| Time |  | 0.27 (0.05)^***^ |  | 0.27 (0.05)^***^ | 0.27 (0.05)^***^ | -0.17 (0.12) |
| Gender (if boys) |  |  |  | -2.41 (0.22)^***^ | -2.42 (0.22)^***^ | -2.65 (0.24)^***^ |
| Self-Esteem |  |  |  | -0.19 (0.01)^***^ | -0.20 (0.01)^***^ | -0.20 (0.01)^***^ |
| Free and Reduced School Meals |  |  |  |  | 1.38 (1.29) | 1.38 (1.29) |
| Ethnicity |  |  |  |  | 0.44 (1.93) | 0.50 (1.94) |
| Year 2 |  |  |  |  | 0.76 (0.34)^*^ | 0.42 (0.37) |
| Year 3 |  |  |  |  | 0.44 (0.32) | -0.15 (0.35) |
| Year 4 |  |  |  |  | 0.20 (0.33) | -0.01 (0.36) |
| Gender (if boys) x Time |  |  |  |  |  | -0.24 (0.10)^*^ |
| Year 2 x Time |  |  |  |  |  | 0.35 (0.15)^*^ |
| Year 3 x Time |  |  |  |  |  | 0.61 (0.14)^***^ |
| Year 4 x Time |  |  |  |  |  | 0.21 (0.25) |
|  | | | | | | |
| Estimates of Covariance Parameters | | |  |  |  |  |
| Repeated Measures | 3.804 (0.134)^***^ | 3.260 (0.163)^***^ |  | 3.272 (0.164)^***^ | 3.272 (0.164)^***^ | 3.271 (0.164)^***^ |
| Individual Intercept | 12.598 (0.697)^***^ | 12.847 (0.835)^***^ |  | 9.013 (0.601)^***^ | 9.065 (0.593)^***^ | 8.998 (0.590)^***^ |
| Individual Covariance Intercept/Slope | 0.699 (0.201)^***^ | -0.777 (0.240)^***^ |  | -0.416 (0.207)^*^ | -0.454 (0.207)^*^ | -0.381 (0.204) |
| Individual Slope |  | 0.473 (0.134)^***^ |  | 0.470 (0.134)^***^ | 0.470 (0.134)^***^ | 0.401 (0.131)^**^ |
| ClassRoom Intercept |  | 0.726 (0.203)^***^ |  | 0.240 (0.123) | 0.081 (0.056) | 0.032 (0.024) |
| ICC | .041 | .043 |  | .019 | .007 | .003 |
| *R^2^* (between-individuals) |  |  |  | .285 | .280 | .286 |
| *R^2^* (between-classes) |  |  |  | .657 | .884 | .954 |
|  | | | | | | |
| *Deviance* (-2_loglikelihood_) | 12216.031 | 12169.778 |  | 11824.908 | 11816.769 | 11790.586 |
| Δ-2LL |  | 46.253^***^ |  | 344.870^***^ | 8.139 | 26.183^***^ |
| Number of estimated parameters | 5 | 7 |  | 9 | 14 | 18 |

*Note.* ^*^*p* < .05; ^**^*p* < .01; ^***^*p* < .001

Supplementary Table 2

*Multilevel Model Analysis Models for Social Withdrawal*

| Parameters | Model 0  Null | Model 1  Level 1: Time |  | Model 2  Level 2: Individual | Model 3  Level 3: Class | Model 4  Cross-Level Interactions  Level 1 and 3 |
| --- | --- | --- | --- | --- | --- | --- |
| Estimates of Fixed Effects | | | | | | |
| Intercept | 5.42 (0.19)^***^ | 5.10 (0.18)^***^ |  | 5.08 (0.16)^***^ | 4.51 (0.26)^***^ | 4.56 (0.26)^***^ |
| Time |  | 0.33 (0.06)^***^ |  | 0.33 (0.06)^***^ | 0.33 (0.06)^***^ | -0.09 (0.11) |
| Gender (if boys) |  |  |  | -0.04 (0.19) | 0.04 (0.19) | 0.02 (0.21) |
| Self-Esteem |  |  |  | -0.25 (0.01)^***^ | -0.24 (0.01)^***^ | -0.24 (0.01)^***^ |
| Free and Reduced School Meals |  |  |  |  | 2.23 (1.29) | 2.17 (1.28) |
| Ethnicity |  |  |  |  | -0.73 (1.94) | -0.76 (1.92) |
| Year 2 |  |  |  |  | 0.42 (0.34) | 0.48 (0.34) |
| Year 3 |  |  |  |  | 0.52 (0.32) | 0.62 (0.32) |
| Year 4 |  |  |  |  | 1.22 (0.33)^***^ | 1.26 (0.33)^***^ |
| Gender (if boys) x Time |  |  |  |  |  | 0.02 (0.09) |
| Year 2 x Time |  |  |  |  |  | 0.45 (0.14)^**^ |
| Year 3 x Time |  |  |  |  |  | 0.76 (0.13)^***^ |
| Year 4 x Time |  |  |  |  |  | 0.21 (0.13) |
|  | | | | | | |
| Estimates of Covariance Parameters | | |  |  |  |  |
| Repeated Measures | 3.442 (0.121)^***^ | 3.254 (0.116)^***^ |  | 3.234 (0.116)^***^ | 3.234 (0.116)^***^ | 3.232 (0.116)^***^ |
| Individual Intercept | 9.398 (0.531)^***^ | 9.471 (0.532)^***^ |  | 5.944 (0.357)^***^ | 5.940 (0.356)^***^ | 5.945 (0.357)^***^ |
| ClassRoom Intercept | 0.946 (0.326)^**^ | 0.679 (0.283)^*^ |  | 0.248 (0.148) | 0.057 (0.105) | 0.050 (0.103) |
| ClassRoom Covariance Intercept/Slope |  | 0.100 (0.070) |  | 0.130 (0.052)^*^ | 0.094 (0.043)^*^ | 0.088 (0.030)^**^ |
| ClassRoom Slope |  | 0.080 (0.036)^*^ |  | 0.078 (0.036)^*^ | 0.079 (0.036)^*^ | 0.012 (0.018) |
| ICC | .069 | .051 |  | .026 | .006 | .005 |
| *R^2^* (between-individuals) |  |  |  | .368 | .368 | .367 |
| *R^2^* (between-classes) |  |  |  | .738 | .940 | .947 |
|  | | | | | | |
| *Deviance* (-2_loglikelihood_) | 11850.875 | 11782.565 |  | 11373.405 | 11360.219 | 11332.370 |
| Δ-2LL |  | 68.310^***^ |  | 409.160^***^ | 13.186^*^ | 27.849^***^ |
| Number of estimated parameters | 5 | 7 |  | 9 | 14 | 18 |

*Note.* ^*^*p* < .05; ^**^*p* < .01; ^***^*p* < .001
